# Metabolomic profiling implicates adiponectin as mediator of a favorable lipoprotein profile associated with NT-proBNP

**DOI:** 10.1186/s12933-018-0765-1

**Published:** 2018-08-28

**Authors:** Annette Masuch, Maik Pietzner, Martin Bahls, Kathrin Budde, Gabi Kastenmüller, Stephanie Zylla, Anna Artati, Jerzy Adamski, Henry Völzke, Marcus Dörr, Stephan B. Felix, Matthias Nauck, Nele Friedrich

**Affiliations:** 1 Institute of Clinical Chemistry and Laboratory Medicine, University Medicine Greifswald, Ferdinand-Sauerbruch-Str, 17475 Greifswald, Germany; 2 German Center for Cardiovascular Research (DZHK e.V.), Partner Site Greifswald, Greifswald, Germany; 3 Department of Internal Medicine B, University Medicine Greifswald, Greifswald, Germany; 4 Institute of Bioinformatics and Systems Biology, Helmholtz Zentrum München, Neuherberg, Germany; 5 Institute of Experimental Genetics, Genome Analysis Center, Helmholtz Zentrum München, Neuherberg, Germany; 6 Lehrstuhl für Experimentelle Genetik, Technische Universität München, Freising-Weihenstephan, Germany; 7 DZD (German Center for Diabetes Research), München-Neuherberg, Germany; 8 Institute for Community Medicine, University Medicine Greifswald, Greifswald, 17475 Germany; 9 DZD (German Center for Diabetes Research), Site Greifswald, Greifswald, 17475 Germany

**Keywords:** Natriuretic peptides, Metabolomics, Adiponectin, Lipoproteins, Population-based study

## Abstract

**Background:**

The N-terminal prohormone of brain natriuretic peptide (NT-proBNP) is an important biomarker for the diagnosis of heart failure. Apart from this and only recently recognized, NT-proBNP levels associate with higher HDL- and lower LDL-cholesterol levels comprising a favorable blood lipid profile. To further examine this observation, the lipoprotein profile in relation to NT-proBNP was examined in-depth by proton nuclear magnetic resonance spectroscopy (^1^H-NMR). We complemented this investigation with a state-of-the-art untargeted metabolomics approach.

**Methods:**

Lipoprotein particles were determined by ^1^H-NMR spectroscopy in 872 subjects without self-reported diabetes from the population-based Study of Health in Pomerania (SHIP)-TREND with available NT-proBNP measurements. Comprehensive metabolomics data for plasma and urine samples were obtained. Linear regression models were performed to assess the associations between serum concentrations of NT-proBNP and the metabolites/lipoprotein particles measured in plasma or urine.

**Results:**

An increase in serum NT-proBNP was associated with a benefical lipoprotein profile, including a decrease in VLDL, IDL and LDL-particles along with an increase in large HDL particles. These findings were replicated in a second independent cohort. Serum concentrations of NT-proBNP showed significant inverse associations with seven plasma metabolites while associations with 39 urinary metabolites, mostly comprising amino acids and related intermediates, were identified. Mediation analyses revealed adiponection as mediating factor for the associations observed with lipoproteins particles.

**Conclusions:**

Most of the metabolic changes associated with NT-proBNP implicate significant influence on the blood lipid profile besides vasodilatory and the diuretic action of BNP signaling. Our data suggest that the more favorable lipoprotein profile as associated with elevated NT-proBNP concentrations in mainly cardiac healthy individuals might relate to adiponectin signaling indicating even indirect cardio-protective effects for NT-proBNP.

**Electronic supplementary material:**

The online version of this article (10.1186/s12933-018-0765-1) contains supplementary material, which is available to authorized users.

## Introduction

The N-terminal fragment of the prohormone of brain natriuretic peptide (NT-proBNP) plays a central role as a biomarker in the diagnosis of heart failure (HF), a syndrome resulting in reduced cardiac output and/or elevated intracardiac pressure at rest or during stress [1]. Elevated levels of NT-proBNP are correlated with increased ventricular mass, another hallmark of chronic HF, and identify pulmonary hypertension [2]. NT-proBNP is the biologically inactive product resulting from the cleavage process which deliberates and activates BNP from proBNP [3, 4]. BNP is produced and released in response to distension or wall stress of the heart and is known to have essential functions in water balance and blood pressure by inducing vasodilation and thereby acting antagonistically to angiotensin II. Further, BNP increases endothelial permeability, directly inhibits aldosterone synthesis and the release of renin, and induces diuresis and natriuresis in the kidney [3, 5]. Recent evidence indicates that natriuretic peptides possess functions beyond cardiovascular homeostasis and relates them to the function and development of adipose tissue [6, 7]. In line with the assumption of an interrelation between body fat and the natriuretic hormone system, higher BNP levels within the reference range were associated with a favorable distribution of adipose tissue meaning lower visceral and liver fat and increased lower-body fat in participants of the DALLAS Heart Study [8]. NT-proBNP and BNP levels are inversely correlated with BMI in the general population and obese individuals have typically lower BNP concentrations independent of cardiovascular disease (CVD) [9, 10]. A recent study further revealed that BNP within the reference range (< 100 ng/L) was inversely correlated with total cholesterol, non-HDL cholesterol, and non-fasting triglycerides representing a favorable lipoprotein profile confirming an observation from a number of previous studies [11–13]. In contrast, in CVD—for which dyslipidemia is a well-known risk factor [14]—NT-proBNP or BNP levels are typically elevated. Importantly, the relation between NT-proBNP and the lipoprotein profile was also observable for concentrations above the reference limit and in the very elderly [15, 16].

In the disease setting, increased NT-proBNP or BNP levels reflect the attempt of the body to restore homeostasis in response to increased intracardiac pressure and/or reduced cardiac output. Thus, BNP is elevated secondary to cardiac dysfunction and independent of adipose tissue function. Overall, NT-proBNP is involved in very different and independent physiologic processes including cardiovascular homeostasis and adipose tissue function.

In order to improve the understanding of NT-proBNP beyond cardiac dysfunction and to explore the relation to lipoprotein metabolism, the present study aimed to examine the associations of NT-proBNP plasma concentrations with the metabolic profile of a large population. As previous studies on NT-proBNP found adiponectin to be associated in a similar manner with a favorable lipoprotein profile [17, 18], a mediation analysis was performed. For this purpose, in-depth analyses of the blood lipid profile associated with NT-proBNP were done using highly resolved lipoprotein particle measures obtained by proton nuclear magnetic resonance (^1^H-NMR) spectroscopy. In order to further broaden the view on possible metabolic implications of NT-proBNP concentrations we complemented these analyses with an untargeted metabolomics approach based on mass spectrometry (MS) and ^1^H-NMR spectroscopy using plasma and urine samples from the same individuals.

## Materials and methods

### Study population—discovery cohort

The Study of Health in Pomerania (SHIP-TREND) is a population-based study located in West Pomerania, a rural region in north-east Germany [19]. A stratified (age, sex and city/county of residence) random sample of 8826 adults aged 20–79 years was drawn from population registries. Sample selection was facilitated by centralization of local population registries in the Federal State of Mecklenburg-West Pomerania. Baseline examinations were conducted between 2008 and 2012. In total, 4420 subjects chose to participate (50.1% response).

For a subsample of 1000 subjects without self-reported diabetes plasma and urine metabolomics data based on MS and ^1^H-NMR were available. After excluding subjects with missing values in serum NT-proBNP measurements, confounding variables or echocardiography the final study sample comprised 872 individuals. Please see below for specification of variables and confounders (Methods, Statistical analysis).

### Study population—replication cohort

Associations between NT-proBNP and lipoprotein particle data obtained by ^1^H-NMR spectroscopy were replicated in the 10 year follow-up of the independent SHIP cohort (SHIP-2) [19]. After application of the same exclusion criteria as outlined above 1420 out of 2333 participants were available for replication. A brief description of the sampling procedure and the study population is given in the Supplemental Information (Additional file 1: Table S1).

#### Ethics, consent and permissions

All participants gave written informed consent before taking part in the study. The study was approved by the local ethics committee and conformed to the principles of the declaration of Helsinki. SHIP data are publically available for scientific and quality control purposes. Data usage can be applied for via http://www.community-medicine.de.

### Laboratory measurements and phenotypic characterization

Smoking status (current, former or never smokers), daily alcohol consumption and physical activity (≥ 1 h training a week) were assessed using computer-aided personal interviews. Waist circumference (WC) was measured to the nearest 0.1 cm using an inelastic tape midway between the lower rib margin and the iliac crest in the horizontal plane.

Fasting blood samples were taken from the cubital vein of participants in the supine position between 7.00 and 13.00 h. In the same time span spot urine samples were taken. All samples were either analyzed immediately or stored at − 80 °C. Blood lipids (total cholesterol, high-density [HDL] and low-density lipoprotein [LDL] cholesterol, triglycerides), serum enzymatic activity concentration of alanine amino transferase (ALT) and serum NT-proBNP were measured by standard methods (Dimension VISTA, Siemens Healthcare Diagnostics, Eschborn, Germany). Serum adiponectin levels were determined using an enzyme-linked immunosorbent assay (Mediagnost, Germany). The inter-assay coefficients of variation were 6.75% and 6.18% for low and high concentrations, respectively. With respect to NT-proBNP, the coefficients of variation were 2.5% at low, 2.3% at medium, and 3.6% at high concentrations of control material, respectively.

Serum creatinine was measured using an enzymatic assay (Dimension VISTA, Siemens Healthcare Diagnostics, Eschborn, Germany) and subsequently the estimated glomerular filtration rate (eGFR) was calculated using the CKD-EPI equation [20].

### Echocardiography

Two-dimensional, M-Mode and Doppler echocardiography were performed using the Vingmed CFM 800A system (GE Medical Systems, Waukesha, USA) as described in detail elsewhere [21]. Measurements of LV end-diastolic and end-systolic diameter (LVD, LVS) were performed according to the guidelines of the American Society of Echocardiography [22]. LV ejection fraction (EF) were calculated following the formula below according to the guidelines of the American Society of Echocardiography [23]: EF (%) = (LVDV - LVSV)/LVDV.

### Metabolomics measurements

A detailed description of all applied measurement techniques is given in the Additional file 1. Briefly, four different approaches were combined in the discovery cohort: (1) non-targeted MS-based profiling of plasma and urine samples as reported previously [24] (2) targeted MS-based profiling of plasma samples using the AbsoluteIDQ p180 Kit (BIOCRATES LifeSciences AG, Innsbruck, Austria) (3) ^1^H-NMR-based profiling of urine samples as reported previously [25] and (4) ^1^H-NMR-based profiling of plasma samples to derive lipoprotein particles.

After quality control and pre-processing (see Additional file 1) 613 plasma and 587 urine metabolites were available for statistical analyses. Note that some of these could not be unambiguously assigned to a chemical identity and are referred to hereafter with the notation “X” followed by a unique number. Data on lipoprotein particles comprise 117 measures describing the gradient from very-low-density lipoprotein (VLDL) particles to HDL particles, including their triglycerides, cholesterol, free cholesterol, phospholipid as well as apolipoprotein B (ApoB), A1 (Apo-A1) and A2 (Apo-A2) content.

Metabolomics data in SHIP-2, i.e. the replication cohort, were restricted to ^1^H-NMR-based profiling of plasma samples with the same approach as described above for SHIP-TREND.

### Statistical analyses

Continuous data are expressed as median (25th; 75th quartile) and nominal data are expressed as percentage. For bivariate analyses, the Kruskal–Wallis test (continuous data) or χ^2^ test (nominal data) was used to compare women and men. Linear regression models were performed to assess the associations between serum concentrations of NT-proBNP and the metabolites measured in plasma and urine (dependent variables). To stabilize variances in linear regression analyses NT-proBNP concentrations and metabolite levels were log-transformed yielding a clear improvement towards normality. Adjustment in linear regression analysis comprised age, sex, WC, smoking, physical activity, eGFR, and ALT levels. A possible modifying effect of sex was tested by inclusion of an interaction term in the linear model. Further, sensitivity analyses were done excluding subjects who reported intake of lipid lowering medication (ATC code C10; N = 59). Possible non-linear associations between NT-proBNP and metabolites/lipoproteins, e.g. achievement of a plateau phase, were tested by means of restricted cubic splines with three knots [26]. To account for multiple testing, we adjusted the p-values from regression analysis by controlling the false discovery rate (FDR) at 5% using the Benjamini–Hochberg procedure. Integration of multi-fluid data was achieved by computation of metabolic networks using Gaussian graphical modelling (GGM). The procedure is outlined in the Additional file 1.

As literature search suggested a possible mediating effect of adiponectin on the association between NT-proBNP and lipoprotein measures, we performed mediation analyses as implemented in the *R* package *mediate*. We defined a significant mediation if the p value was < 0.01 (using 2000 bootstrap samples) and at least 10% of the association was mediated. Statistical analyses were performed using SAS version 9.4 (SAS statistical software, version 9.4, SAS Institute, Inc; NC, USA) and R 3.1.1 (R Foundation for statistical computing, version 3.1.1, Vienna, Austria).

## Results

### Study population

Age, eGFR, and physical activity were comparable between men and women (Table 1). However, women showed a more benficial lipid profile, mainly due to higher HDL-cholesterol values, compared to men. NT-proBNP levels were clearly higher in women despite having a slightly better median ejection fraction.

**Table 1:** General characteristics of the study population

Characteristic	Men (n = 373)	Women (n = 499)	p*
Age (years)	49 (38; 60)	49 (40; 59)	0.94
Smoking (%)			< 0.01
Never smoker	32.7	49.7	
Former smoker	44.8	28.5	
Current smoker	25.5	21.8	
Physically active (%)	74.3	73.7	0.93
Waist circumference (cm)	93 (85; 101)	80 (73; 89)	< 0.01
NT-probnp (ng/L)	32 (19; 55)	68 (41; 124)	< 0.01
Triglycerides (mmol/L)	1.27 (0.92; 1.88)	1.13 (0.81; 1.60)	< 0.01
HDL-cholesterol (mmol/L)	1.29 (1.11; 1.49)	1.58 (1.35; 1.83)	< 0.01
LDL-cholesterol (mmol/L)	3.35 (2.73; 3.96)	3.31 (2.72; 3.95)	0.55
Total cholesterol (mmol/L)	5.3 (4.5; 6.0)	5.5 (4.9; 6.2)	< 0.01
Egfr (mL/min/1.73 m^2^)	98 (89; 108)	98 (86; 107)	0.28
ALT (µkatal/L)	0.46 (0.35; 0.64)	0.30 (0.24; 0.41)	< 0.01
Ejection fraction (%)	72 (65; 77)	73 (68; 79)	< 0.01
Self-reported HF (%)	1.9%	2%	1.00
Adiponectin (ng/mL)	5644 (3814; 7782)	8851 (6333; 12,102)	< 0.01

Continuous data are expressed as median (25th percentile; 75th percentile); nominal data are given as percentages. * χ^2^-test (nominal data) or Mann–Whitney-U test (interval data) were performed to compare men and women

*HDL* high density lipoprotein, *LDL* low-density lipoprotein, *NT-proBNP* N-terminal prohormone of brain natriuretic peptide, *eGFR* estimated glomerular filtration rate, *ALT* alanine aminotransferase, *HF* heart failure

As no obvious sex-interactions (FDR < 0.2) were observed we did not stratify the analyses despite the strong differences in NT-proBNP concentrations between men and women.

### Plasma lipoproteins

**Fig. 1:**
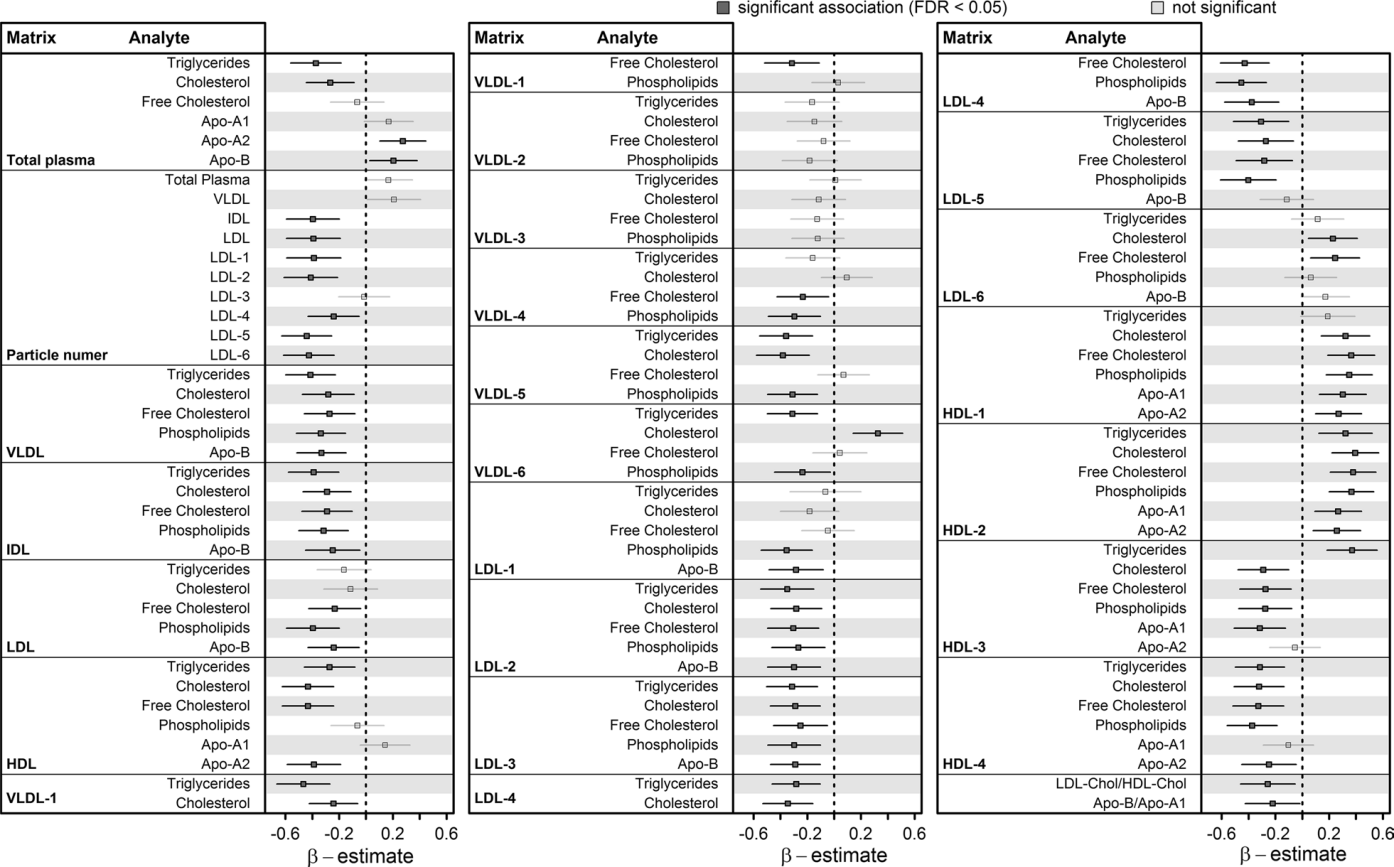
Associations of NT-proBNP concentrations with the whole-range of lipoprotein particle measures received by ^1^H-NMR spectroscopy. Depicted are beta-estimates with 95%-confidence intervals from linear regression analysis for lipoprotein subclasses and derived variables with serum NT-proBNP concentrations. The first column of each block contains the matrix, e.g., the specific particle, and the second column contains the analyte determined, e.g., the cholesterol content. Significant associations (controlling the false discovery rate (FDR) at 5%) are indicated by dark grey. *VLDL* very low-density lipoprotein, *IDL* intermediate-density lipoprotein, *LDL* low-density lipoprotein, *HDL* high-density lipoprotein, *Apo* apolipoprotein

Figure 1 presents the results of NT-proBNP concentrations associated with the whole range of plasma lipoprotein particles and contents. An increase in serum NT-proBNP was associated with a beneficial lipoprotein profile, including less VLDL, intermediate-density lipoprotein (IDL), and LDL-particles along with higher large HDL particle measures and lower small dense HDL particle measures (Fig. 1). Even plasma cholesterol and triglycerides were significantly inversely associated with NT-proBNP. In particular, an inverse association with measures of small dense LDL-particles was observable. The ratio between total Apo-B and Apo-A1 reflected the beneficial shift in the lipid profile with higher NT-proBNP as well (Fig. 1).

The exclusion of subjects taking lipid-lowering medication did not lead to any substantial changes in the association results with respect to the lipoprotein profile (Additional file 1: Fig. S1). Further, with the exception of the association with HDL2-particles, all associations could be replicated in the independent SHIP-2 cohort (Additional file 1: Fig. S2).

### Plasma and urine metabolome

**Fig. 2:**
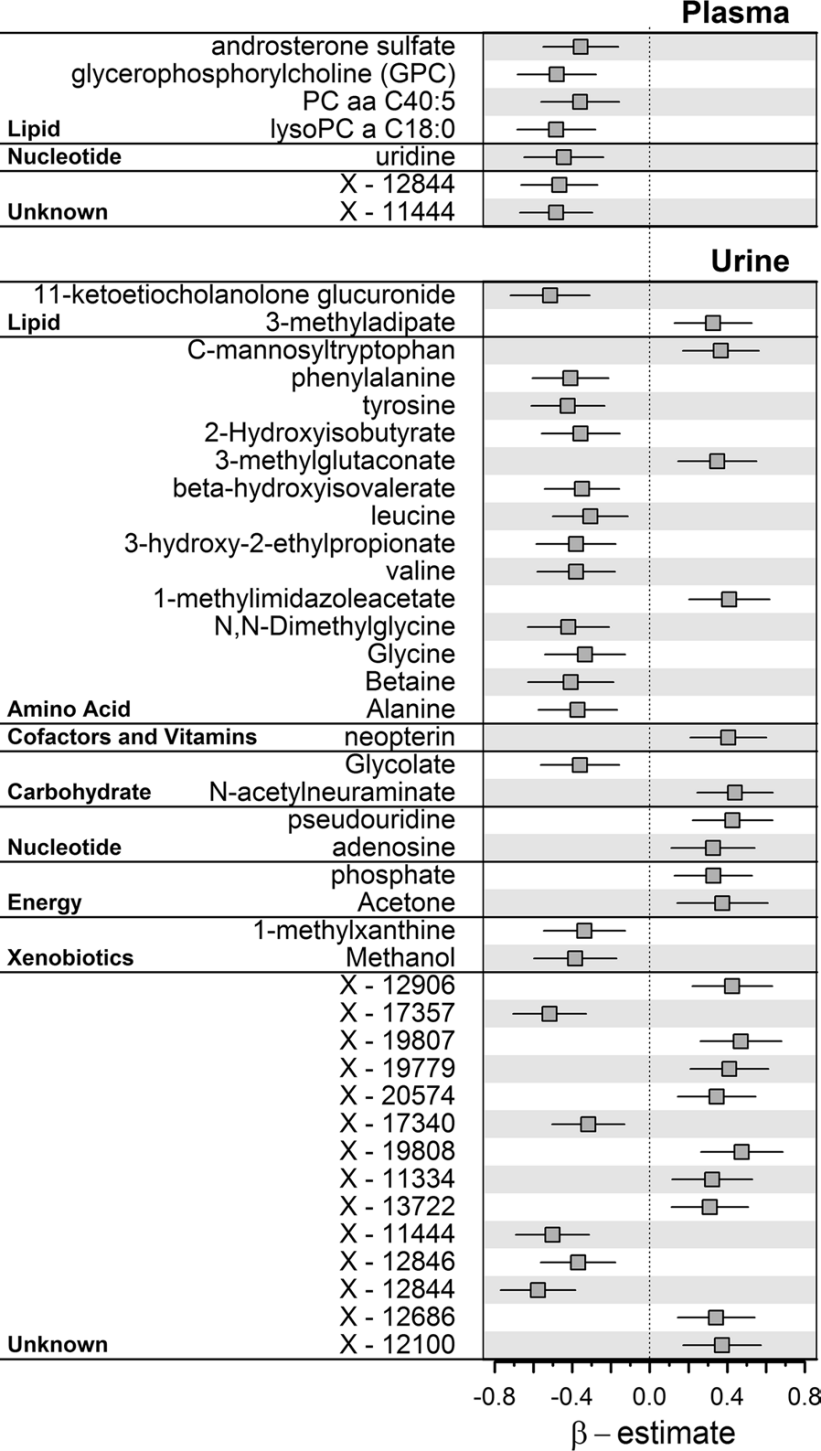
Beta-estimates (points) with 95%-confidence intervals (lines) from linear regression analysis for plasma and urine metabolites significantly associated (controlling the false discovery rate at 5%) with serum NT-proBNP concentrations

Serum concentrations of NT-proBNP showed solely significant inverse associations with seven plasma metabolites (Fig. 2). Those included androsterone sulfate, glycerophosphocholine (GPC), the diacyl phosphatidylcholine (PC aa XX:Y) PC aaC40:5, the lysophosphatidylcholine (lysoPC) C18:0, uridine and the two unknown compounds X-12844 and X-11444.

A total of 39 urine metabolites showed significant associations with NTproBNP (Fig. 2). One signature comprised amino acid species, in particular inverse associations with aromatic and branched-chain amino acids (BCAA) and related degradation intermediates (Fig. 2). Only C-mannosyltryptophan, 3-methylglutaconate, and 1-methylimidazoleacetate were an exception showing positive associations. Further inverse associations were observed for urinary levels of 11-ketoetiocholanolone glucuronide, glycolate, 1-methylxanthine, and methanol as well as several unknown compounds. Among the latter, the strong inverse associations with X-11444 and X-12844 seen in plasma were replicated in urine (Fig. 2). Positive associations with serum NT-proBNP comprised neopterin, *N*-acetylneuraminate, pseudouridine, adenosine, phosphate, and acetone as well as several unknown compounds.

#### Integration of multifluid data and unknown parameters

**Fig. 3:**
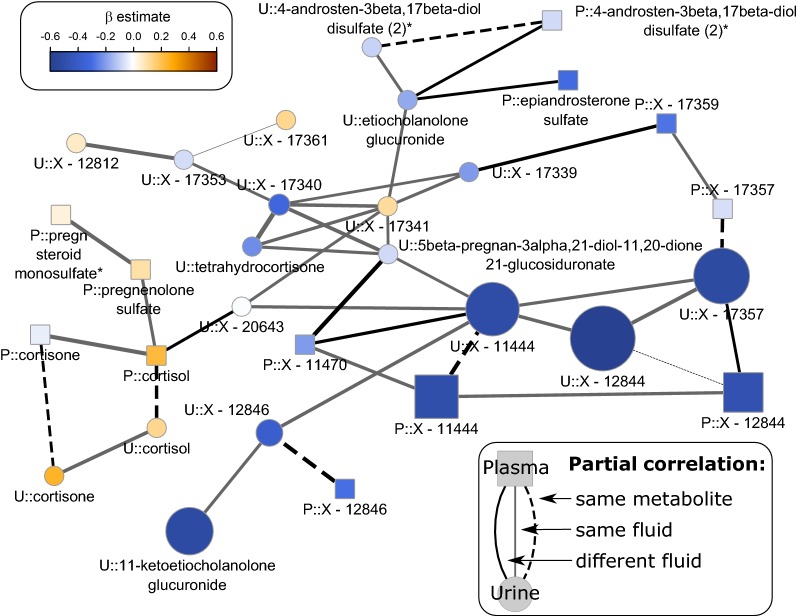
Subnetwork from the Gaussian graphical model to reconstruct metabolite dependencies with a particular focus on adrenal steroids. Node size is determined by −log10 (FDR-value) from linear regression analyses with NT-proBNP as exposure and colors indicate effect directions—blue = inverse and orange = positive. Suffix P:: and U:: indicate plasma and urine metabolites, respectively

To integrate urine and plasma metabolome data GGMs were computed to reconstruct physiological dependencies in a data driven manner. After discarding metabolites with more than 20% missing values, the final GGM consisted of 785 nodes and 1065 edges. Subsequently, we projected the results, i.e. beta estimates and p-values, from linear regression analyses on the metabolite network by increasing node size (as −log10(p-value)) and color gradient (beta estimate ranging from blue, strong inverse association, to orange, strong positive association). Direct neighborhood within the GGM is a strong indicator of biochemical, and hence physiological, dependencies between metabolites [27]. By visual inspection of such a modified GGM we could integrate the unknown metabolites X-11444 and 12844, which were strongly associated with NT-proBNP in both fluids, together with 11-ketoetiocholanolone glucuronide into a steroid metabolite cascade (Fig. 3).

### Mediation by serum adiponectin concentrations

**Fig. 4:**
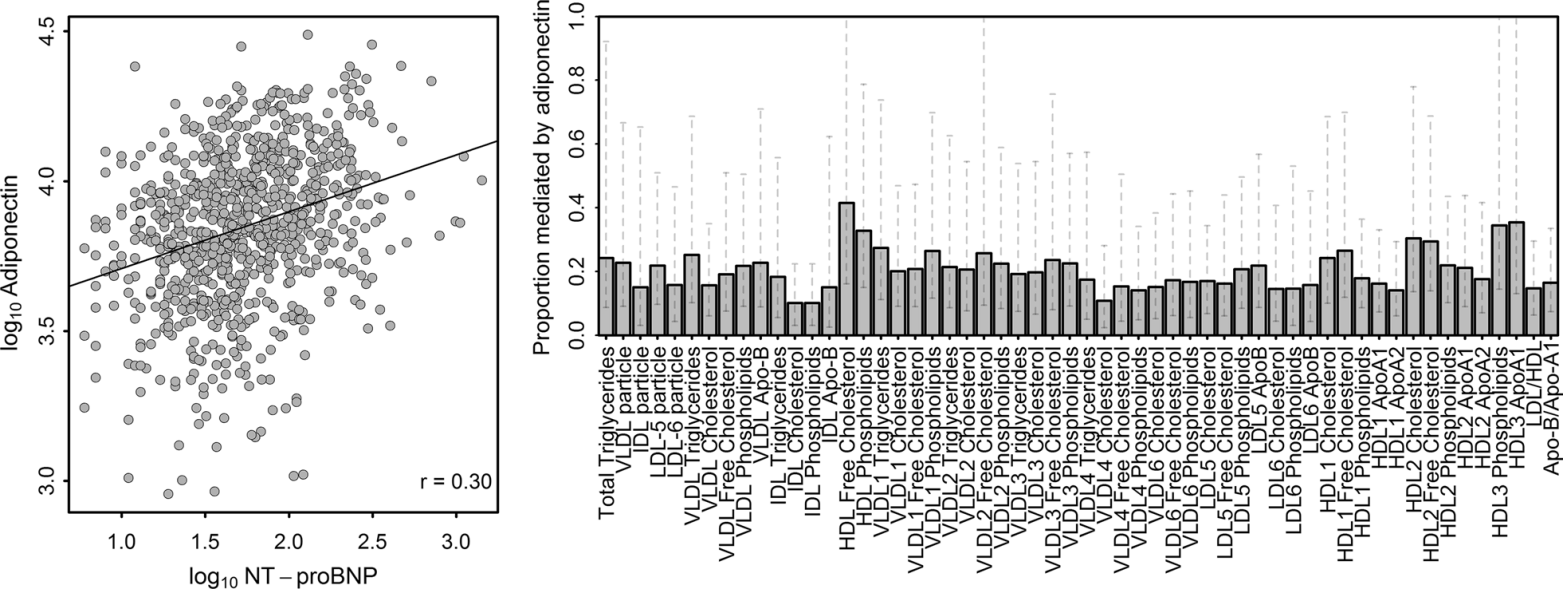
*Left:* Scatterplot of serum N-terminal prohormone of brain natriuretic peptide (NT-proBNP) and adiponectin concentrations on a logarithmized scale. The black line indicates a simple linear regression fit. *Right:* Proportion mediated (bars) with 95% confidence intervals by serum adiponectin concentrations for lipoprotein subclass measures significantly associated with serum NT-proBNP in linear regression analyses

Plasma adiponectin showed a significant correlation with serum NT-proBNP (r = 0.30; p < 0.01; Fig. 4). Mediation analyses revealed a sustainable influence of serum adiponectin on almost all associations between serum NT-proBNP and lipoprotein subclass measures (Fig. 4) reaching a median proportion mediated of about 0.2. In particular, associations with VLDL, small dense LDL as well as large HDL particles were affected. The association with total cholesterol was not affected. Notably, with the exception of the urine unknown X-17340 none of the small molecule associations was affected.

## Discussion

The present study examined the metabolic fingerprint of NT-proBNP in a sample from the general population without self-reported diabetes to decipher underlying metabolic reasons for the observed associations of higher NT-proBNP concentrations with the presence of a beneficial lipoprotein profile. Almost the whole density gradient of the lipoprotein profile was positively shifted which was observed in two independent cohorts. Using a non-targeted metabolomics approach revealed only few metabolites in plasma but several metabolites in urine to be associated with NT-proBNP.

For the comprehensive understanding of NT-proBNP associated metabolites, the physiologic role of BNP as the biologically active mature peptide after cleavage from NT-proBNP has to be considered. NT-proBNP and BNP are released in equimolar amounts but have different plasma half-life with 120 min and 20 min, respectively [28]. BNP as a member of the natriuretic peptide hormone family modulates diverse biological functions, including regulation of blood pressure and water balance, which were partially reflected by the metabolic signature seen with respect to NT-proBNP. As mainly cardiac healthy individuals were examined, the observed associations can be considered independent of severe cardiac dysfunction. Importantly, the present results indicate that the relation between NT-proBNP and a beneficial lipoprotein profile might be mediated by adiponectin.

### Beneficial lipoprotein profile

In general, low HDL-cholesterol and high LDL-cholesterol are significantly associated with the risk to develop CVD [29]. Notably, besides being a marker of cardiac dysfunction, higher values of NT-proBNP were associated with a more beneficial lipoprotein profile among our apparently healthy study population as well as in a population of more advanced age (SHIP-2). Our findings are in line with an observation among 680 elderly volunteers with NT-proBNP levels below 100 ng/L exhibiting an inverse association between BNP and non-HDL cholesterol [11]. Likewise, Takeuchi et al. observed a strong inverse correlation between serum BNP concentrations and total cholesterol in a population of patients [30]. In older adults free of cardiovascular or kidney disease increased levels of NT-proBNP related to lower LDL-cholesterol and higher HDL-cholesterol [17]. Notably, adiponectin levels were similarly associated with a favorable lipoprotein profile in this study, which was also reported by others [18]. Also serum adiponectin was linked to serum (NT-pro)BNP [18, 31]. Indeed, with respect to a possible mechanism accounting for these observations, the mediation of the association of NT-proBNP with a beneficial lipoprotein profile via adiponectin has been shown in the present study.

### Adiponectin in the cardiovascular system

**Fig. 5:**
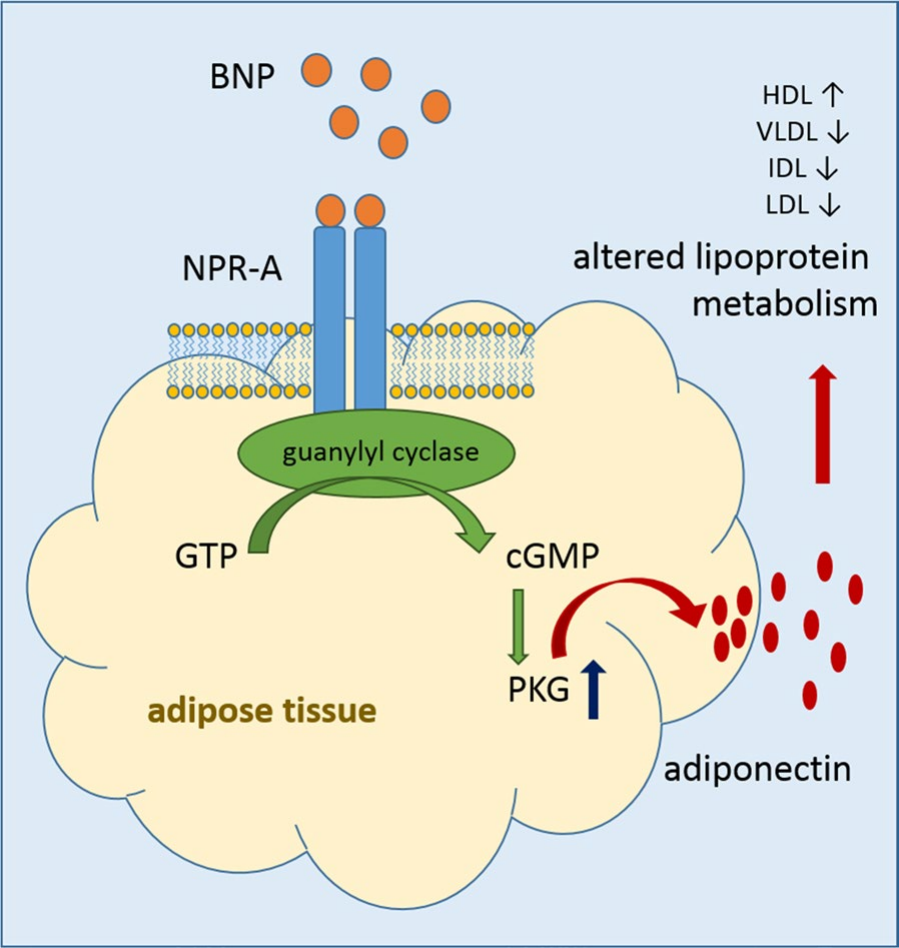
Proposed pathway based on the analysis of the metabolome and lipidome data. BNP binds to NPR-A in adipose tissues leading to activation of guanylyl cyclase catalyzing the formation of cGMP from GTP. PKG is activated promoting the release of adiponectin which in turn affects lipoprotein metabolism shifting the lipoprotein profile towards a more favorable status. *BNP* brain natriuretic peptide; *NPR-A* natriuretic peptide receptor A, *GTP* guanosine triphosphate, *cGMP* cyclic guanosine monophosphate, *PKG* cGMP-dependent protein kinase, *HDL* high-density lipoprotein, *VLDL* very low-density lipoprotein, *IDL* intermediate-density lipoprotein, *LDL* low-density lipoprotein

Adiponectin is an adipokine which is known to be almost exclusively produced and secreted by adipocytes. However, in addition to adipocytes, various other cell types and tissues secrete adiponectin. Besides its main metabolic role in glucose homeostasis, adiponectin is also implicated in many other metabolic and inflammatory functions, including for instance tissue regeneration [32] and proliferation and function of sebocytes [33]. Considering a relation between BNP and adiponectin, it has to be noted that perivascular adipose tissue (PVAT) has significant influence on the vascular tone releasing anti-contractile factors, one of them being adiponectin. The overall function of PVAT with respect to vasodilation seems to be strongly dependent on cGMP-dependent protein kinase (PKG) [34] which is also the effector kinase activated in response to BNP-binding to the natriuretic peptide receptor A (NPR-A) [2]. In view of these complementary vasodilator effects described for of BNP and PVAT and comprising both the activation of PKG, it is tempting to assume that increased BNP may directly relate to enhanced adiponectin release from adipocytes. Indeed, Tsukamoto et al. showed in cultured human adipocytes increased adiponectin gene expression and adiponectin secretion in response to natriuretic peptide treatment and the study also demonstrated increased plasma levels of adiponectin in patients with congestive HF receiving atrial natriuretic peptide [35]. Adiponectin in turn alters lipoprotein metabolism on different levels, e.g. by suppressing hepatic lipase activity [36] or promoting activation of lipoprotein lipase [37, 38]. Taken together, a potential pathway that may contribute to the improved lipoprotein profile associated with NT-proBNP might rely on BNP signaling on adipocytes and NPR-A activation promoting PKG elevation followed by enhanced adiponectin release which finally influences lipoprotein metabolism (Fig. 5).

Numerous studies uncovered various functions of adiponectin which further support its positive metabolic influence. Oxidative stress plays a pivotal role in inflammatory processes and in turn contributes to development and progression of a number of diseases including CVD, type 2 diabetes mellitus (T2DM) and neurodegenerative diseases [39]. In this context, adiponectin has been reported to reduce production of reactive oxygen species preventing damage due to free radicals [40]. In line with this, in patients with T2DM levels of circulating adiponectin was lower in serum compared to normoglycemic subjects and vascular free radical formation related to NADPH oxidase was increased, which in turn activated PVAT to increase adiponectin expression in response to increased free radical formation [41]. Higher circulating adiponectin levels were also associated with a beneficial development of heart rate variability over 5 years in T2DM representing improved cardiovascular autonomic function [42]. Comparing a native Japanese population with Japanese-American people revealed lower serum adiponectin and increased measures of inflammation in the later indicating that westernization of lifestyle may affect adiponectin which in turn might promote development of insulin resistance [43]. Strengthening the role of adiponectin in T2DM, an increase of circulating adiponectin in response to anti-diabetic treatment has been reported [44]. However and in contrast to these reports, adiponectin has been found to be positively associated with increased cardiovascular mortality rate. The reasons for this observations remain to be unraveled [45].

### Volume factors, kidney function and link to inflammation

In order to try to understand underlying metabolic processes explaining the observations above, associations between NT-proBNP and the plasma and urine metabolome were investigated. Several associated metabolites, e.g. uridine in plasma as well as adenosine or 1-methylimidazole in urine point towards the vasodilatory role of BNP. For instance, uridine can induce vasodilation by activation of purinergic P2Y receptors leading to release of nitric oxide, a well-known vasodilator [46]. The metabolite GPC is an abundant osmolyte in renal medulla cells and was inversely associated with NT-proBNP in plasma. Similarly, betaine acts also as osmolyte and accumulates together with GPC, sorbitol, and inositol to sufficiently counterbalance osmolality [47]. The inverse association of plasma GPC as well as urinary betaine with NT-proBNP appears to be a physiologic consequence of BNP signaling with respect to its role in diuresis.

Despite controlling for kidney function by means of the eGFR as surrogate marker, two metabolites in urine previously reported as surrogates for glomerular filtration in plasma [48], namely pseudouridine and C-mannosyltryptophan, were positively associated with NT-proBNP in linear regression analyses.

In previous work we identified urinary C-mannosyltryptophan as important predictor of an advanced inflammatory state in almost the same individuals as in the present investigation [49]. In this context the positive association of NT-proBNP with urinary neopterin might be of particular interest. Neopterin belongs to the group of pteridines and is derived from guanosine triphosphate in activated monocytes, macrophages, dendritic cells and endothelial cells, but also to a lesser extent in renal epithelial cells. Typically, neopterin is considered a marker of enhanced immune activation [50]. Neopterin in its unchanged form is excreted via the kidney and its levels correlate with the extent of disease or infection [51]. In addition, Grammer et al. identified NT-proBNP and also the eGFR as strongest predictors for neopterin serum concentration [50] linking neopterin to kidney function. Interestingly, also circulating adiponectin seems to be related with neopterin concentrations. Accordingly, a small observational study has observed that individuals with low HDL-cholesterol are characterized by low circulating adiponectin and high neopterin compared to individuals with high HDL-cholesterol This was interpreted as inflammatory phenotype in individuals with low HDL-cholesterol [52] linking adiponectin also to inflammation.

Estimation of the glomerular filtration rate using serum creatinine suffers from a number of drawbacks, e.g. difference in muscle mass affecting creatinine release, so we could not rule out residual confounding via capturing the true glomerular filtration rate of the participants. However, the metabolite signature comprising urine levels of pseudouridine, C-mannosyltryptophan and neopterin links serum NT-proBNP to impaired kidney function rather than cardiac dysfunction.

### Steroidogenesis in the adrenal glands

A cluster of adrenal-derived steroids and putative metabolites thereof (Fig. 2) was inversely associated with NT-proBNP. It has been shown by Liang and coworkers in primary human adrenocortical cells, that BNP inhibited gene expression related to the angiotensin II induced steroidogenesis, including the de novo synthesis of cholesterol and other steps from cholesterol supply till steroid formation, namely cortisol, in mitochondria [53]. In line with this, NT-proBNP associated urinary cortisol metabolites indicate a potential shift in cortisol metabolism favoring activation or diminished degradation, as 11-ketoetiocholanolone glucuronide and the unknown X-12844, recently identified as tetrahydrocortisone glucuronide [54], were inversely associated with NT-proBNP. In line with the described inhibition of BNP on angiotensin II induced steroidogenesis, the strong inverse association with NT-proBNP may be a consequence of affected cortisol synthesis. Of note, adrenal glands are embedded into periadrenal adipose tissue and adiponectin receptors are expressed in human adrenal cortex [55]. Moreover, adiponectin itself was reported to affect adrenal steroidogenesis [56]. Thus, the observed associations with cortisol metabolites may implicate again that adiponectin may play a role in the observed associations. No association with plasma cortisol became obvious emphasizing local influence on cortisol metabolism as potential mechanism.

### Natriuretic peptides and energy consumption

A prominent molecular signature related to NT-proBNP was a diminished urinary excretion of BCAAs and related intermediates. In line with the fact that obese individuals have lower levels of NT-proBNP independent of cardiac health [10], elevated circulating BCAA concentrations were found to be associated with obesity and insulin resistance, and have been linked to metabolic syndrome and CVD [57]. In regard of the prominent role of skeletal muscle as well as adipose tissue in BCAA catabolism [58], starting with the conversion of BCAA into their respective α-keto acids within mitochondria, it might be conceivable that an inverse association of BCAA metabolites in urine with NT-proBNP is also related to BNP-signaling dependent increase in mitochondrial biogenesis. BNP-signaling induces mitochondrial biogenesis in skeletal muscles and enhances mitochondrial capacity of β-oxidation [59]. An increase in the number of mitochondria would in turn improve degradation of BCAAs and hence limit their urinary excretion. Notably, also adiponectin has been reported to induce mitochondrial biogenesis in skeletal muscle [60] posing again a relation between BNP and this adipokine. In this context, adiponectin has been shown to sequentially stimulate activity of the AMP-activated protein kinase (AMPK), p38 mitogen-activated protein kinase (MAPK) and peroxisome proliferator-activated receptor (PPAR) α thereby enhancing fatty acid oxidation in skeletal muscle [61].

Furthermore, natriuretic peptides have potent lipolytic effects in humans as observed upon intravenous infusion as well as subcutaneous infusion into abdominal adipose tissue [7, 62]. In line with this, NPR-A activation in adipocytes increased energy expenditure and heat production (thermogenesis) in mice [62, 63]. Activation of brown adipose tissue is similarly inducible by cold-exposure. It has to be noted, that activation of brown adipose tissue by cold-exposure enhanced the triglyceride clearance from circulation, while HDL-cholesterol abundance increased and was sufficient to correct hyperlipidemia [64]. As natriuretic peptide signaling and cold-exposure seem to share effects on lipolysis and thermogenesis, it may be conceivable that also changes in the HDL metabolism, as observed in rodents and lean but not obese humans upon cold-exposure [65], may be among these shared effects providing a further possible explanation for the observed associations.

### Strengths and limitations

The major strength of our study is the comprehensive profiling of the metabolome content of plasma and urine samples using the complementary techniques MS and ^1^H-NMR allowing us to screen for potential molecular signatures of alterations in NT-proBNP on a large-scale. The presence of only few individuals with self-reported HF minimized confounding of the associations by cardiac dysfunction but also limits our observations with respect to clinical application in this context. Nevertheless, the replication of the relation with a favorable lipid profile in an independent population strongly argues to augment the role of (NT-pro)BNP (and circulating adiponectin) concentrations in relation to cardiac dysfunction.

## Conclusion

Considering the controversies that exist with respect to the paradox of low NT-proBNP in obese individuals and the known observation of increased NT-proBNP accompanying cardiac dysfunction for which obesity is a major risk factor, it can be noted from the present study that both observations are likely independent of each other. The present study demonstrates that NT-proBNP is positively associated with a beneficial lipoprotein profile covering almost the whole density gradient. As HF patients were almost absent in the present study population, the majority of the observed associations with small molecules likely reflect the physiological consequences of BNP signaling. Overall, the current data suggest a mechanism that, in absence of severe cardiac dysfunction, links higher NT-proBNP concentrations to a more favorable lipoprotein profile involving increased adiponectin release from adipose tissue. Future studies on this issue may further elucidate the underlying molecular mechanisms.

## Additional file

**Additional file 1.** Detailed methods description and supporting results. Population characteristics for the replication population SHIP-2 and the results for the in-depth analysis of the lipoprotein profile are presented. Furthermore, the results of the sensitivity analysis on the influence of lipid lowering drugs on the lipoprotein profile is given.

## Abbreviations

NT-proBNP: N-terminal fragment of the prohormone of brain natriuretic peptide
HF: heart failure
CVD: cardiovascular disease
^1^H-NMR: proton nuclear magnetic resonance
MS: mass spectrometry
SHIP: Study of Health in Pomerania
WC: waist circumference
HDL: high-density lipoprotein
LDL: low-density lipoprotein
ALT: alanine amino transferase
eGFR: estimated glomerular filtration rate
EF: ejection fraction
VLDL: very-low-density lipoprotein
Apo: apolipoprotein
FDR: false discovery rate
GGM: Gaussian graphical modelling
IDL: intermediate-density lipoprotein
GPC: glycerophosphocholine
PC aa XX:Y: diacyl phosphatidylcholine
lysoPC: lysophosphatidylcholine
PVAT: perivascular adipose tissue
PKG: cGMP-dependent protein kinase
NPR-A: natriuretic peptide receptor A
T2DM: type 2 diabetes mellitus
