# Supplementary material for: Metabolomic profiling implicates adiponectin as mediator of a favorable lipoprotein profile associated with NT-proBNP
Source: Cardiovasc Diabetol. 2018 Aug 28;17:120. doi: 10.1186/s12933-018-0765-1 (PMC6112131; doi:10.1186/s12933-018-0765-1)
Supplement: Supplementary file 1 — Additional file 1. Detailed methods description and supporting results. Population characteristics for the replication population SHIP-2 and the results for the in-depth analysis of the lipoprotein profile are presented. Furthermore, the results of the sensitivity analysis on the influence of lipid lowering drugs on the lipoprotein profile is given. [file 12933_2018_765_MOESM1_ESM.docx]

**Additional file**

**Metabolomic profiling implicates adiponectin as mediator of a favorable lipoprotein profile associated with NT-proBNP**

Annette Masuch^1*^, Maik Pietzner^1,2*^,Martin Bahls^2,3^, Kathrin Budde^1,2^, Gabi Kastenmüller^4^, Stephanie Zylla^1,2^, Anna Artati^5^, Jerzy Adamski^5,6,7^, Henry Völzke^2,8,9^, Marcus Dörr^2,3^, Stephan B. Felix^2,3^, Matthias Nauck^1,2^, Nele Friedrich^1,2^

**METHODS**

***Untargeted LC-MS/MS profiling***

*Metabolomics measurements*

Non-targeted metabolomics analysis for metabolic profiling was conducted at the Genome Analysis Center, Helmholtz Zentrum München. Two separate LC-MS/MS analytical methods as previously described, i.e. in positive and in negative ionization modes, were used to detect a broad metabolite panel (1). In this study, samples were divided into two sets according to the biological matrices of the samples, i.e. plasma and urine. On the day of extraction, samples were thawed on ice. Of each sample 100 µL were pipetted into a 96-well plate (2 mL volume per well). In addition to study samples, a pooled human reference plasma sample (Seralab, West Sussex, United Kingdom) and another pooled reference matrix of each sample set (Seralab, West Sussex, United Kingdom) were extracted (1 well and 6 wells, respectively). These samples served as technical replicates throughout the data set to assess process variability. Beside those samples, 100 μL of water was extracted as samples to serve as process blanks (6 wells). Protein was precipitated and the metabolites were extracted with 475 µL methanol, containing four recovery standards to monitor the extraction efficiency. After centrifugation, the supernatant was split into 4 aliquots of 100 µL each onto two 96-well microplates. The first 2 aliquots were used for LC-MS/MS analysis in positive and negative electrospray ionization mode. Two further aliquots were kept as a reserve. The extracts were dried on a TurboVap 96 (Zymark, Sotax, Lörrach, Germany). Prior to LC-MS/MS in positive ion mode, the samples were reconstituted with 0.1% formic acid (50 µL for plasma, 100 µL for urine). Whereas samples analyzed in negative ion mode were reconstituted with 6.5 mM ammonium bicarbonate (50 µL for plasma, 100 µL for urine), pH 8.0. Reconstitution solvents for both ionization modes contained internal standards that allowed monitoring of instrument performance and also served as retention reference markers. To minimize human error, liquid handling was performed by a Hamilton Microlab STAR robot (Hamilton Bonaduz AG, Bonaduz, Switzerland). LC-MS/MS analysis was performed on a linear ion trap LTQ XL mass spectrometer (Thermo Fisher Scientific GmbH, Dreieich, Germany) coupled with a Waters Acquity UPLC system (Waters GmbH, Eschborn, Germany). Two separate columns (2.1 x 100 mm Waters BEH C18, 1.7 µm particle-size) were used either for acidic (solvent A: 0.1% formic acid in water, solvent B: 0.1% formic acid in methanol) and or for basic (A: 6.5 mM ammonium bicarbonate, pH 8.0, B: 6.5 mM ammonium bicarbonate in 95% methanol) mobile phase conditions, optimized for positive and negative electrospray ionization, respectively. After injection of the sample extracts, the columns were developed in a gradient of 99.5% A to 98% B over an 11 min run time at 350 µL/min flow rate. The eluent flow was directly run through the ESI source of the LTQ XL mass spectrometer. The mass spectrometer analysis alternated between MS and data-dependent MS/MS scans using dynamic exclusion and the scan range was from 80-1000 m/z. Metabolites were identified by Metabolon, Inc. from the LC-MS/MS data by automated multiparametric comparison with a proprietary library, containing retention times, m/z ratios, and related adduct/fragment spectra (2). Identification criteria for the detected metabolites are described in Evans *et al.* (1).

*Metabolomics measurements: quality control and normalization of metabolite levels*

To correct for daily variations of platform performance, the raw ion count of each metabolite was rescaled by the respective median value of the run day. Valid estimation of the median was ensured by keeping only metabolites with at least three measured values on more than the half of the run days. This procedure resulted in 475 and 558 metabolites for plasma and urine, respectively, available for the present analysis. 263 metabolites were measured in both bio fluids. We chose probabilistic quotient normalization (PQN) (3) to account for diurnal variation of urine samples, since this procedure was shown to be superior to the common creatinine scaling. For this purpose we calculated a mean-pseudo-spectrum depending on metabolites with measurements for all participants (131 urine metabolites). Subsequently, we calculated a dilution factor as the median quotient between the reference spectrum and each sample. Of note, urine creatinine and the estimated dilution factor were highly correlated (r=0.91, p<0.001) within the present study sample. Afterwards all metabolite levels were log_2_-transformed. Separately for plasma and urine samples we performed multivariate outlier detection using an algorithm proposed by Filzmoser *et al.* (4) as implemented in the *pcout* function within the R package *mvoutlier*. The algorithm provides an outlier score for each sample based on a weighted combination of location and scatter estimations using principle component analysis and the Mahalanobis distance on a robustly scaled data matrix. The default parameters were used for the identification process, except the critical value for the location outliers was set to 4, as it corresponds to a 4 SD exclusion criteria. The minimum score was used as cut-off for outlier identification. As a result 13 and 8 samples from plasma and urine were excluded, respectively.

***Targeted LC-MS/MS profiling***

*Metabolomics measurements*

Targeted metabolomics profiling of the plasma samples was performed using the AbsoluteIDQ p180 Kit (BIOCRATES LifeSciences AG, Innsbruck, Austria, online supplementary methods). 10 µL aliquots of each plasma sample were processed as recommended by the manufacturer. The fully automated assay combined flow injection (FIA) and LC-MS/MS selective detection using MRM pairs and quantifies up to 188 metabolites from 5 different compound classes. Via FIA acyl carnitines, phospho- and sphingolipids were measured in positive ionization mode and the sum of hexoses in negative ionization mode. With a LC-MS/MS analytical method, under the use of an Agilent C18 column, amino acids and biogenic amines were detected. MS analyses were performed on an AB SCIEX 5500 QTrap™ mass spectrometer (AB SCIEX, Darmstadt, Germany) with electrospray ionization combined with a HPLC system (Agilent 1260 Infinity Binary LC, Santa Clara, United States) including a degasser unit, column oven, autosampler and a binary pump. Internal standards (isotope labelled) are partially integrated in the Kit plate for metabolite quantification. After the measurement a pre-processing step, includes peak integration and concentration determination from calibration curves, with Analyst software (Version 1.5.1, AB Sciex, Darmstadt, Germany), data were uploaded into Biocrates MetIDQ software (part of the kit) and the metabolite concentrations were automatically calculated with it.

*Metabolomics measurements: quality control and normalization of metabolite levels*

To account for between-plate variation, a sole sample dependent normalization was performed. To this end for each plate the measured concentrations of the metabolites were divided by the median concentration leading to equal median values for each metabolite on each plate. Subsequently, the median of the plate medians was calculated to reset to the original scale (µM concentrations). No obvious pattern in missing values along the measurement period became obvious. However, only metabolites with at least 20% valid observations were included in the final data sets, resulting in 183 used for subsequent analysis. PCA was performed to detect multivariate outliers. These were defined as samples deviating more than three times the standard deviation (SD) from the mean Mahalanobis distance based on the first ten principle components. As a result, four samples were excluded. Finally, metabolite levels were log_2_-transformed.

***^1^H-NMR profiling***

*Urine measurements*

Prior analysis urine specimens were stored about five years at -80°C. After thawing, urine specimens were centrifuged for 5 min at 3000 x*g* and the supernatant was used for spectroscopic analysis. For this purpose, 450 µL urine were mixed with 50 µL phosphate buffer in order to stabilize the urinary pH at 7.0 (±0.35). The phosphate buffer was prepared with D2O and contained sodium 3-trimethylsilyl-(2,2,3,3-D4)-1-propionate (TSP) as reference. Spectra were recorded at the University Medicine Greifswald, Germany, on a Bruker DRX-400 NMR spectrometer (Bruker BioSpin GmbH, Rheinstetten, Germany) operating at ^1^H frequency of 400.13 MHz and equipped with a 4-mm selective inverse flow probe (FISEI, 120 µL active volume) with z-gradient. Specimens were automatically delivered to the spectrometer via flow injection. The acquisition temperature was set to 300°K. A standard one-dimensional ^1^H-NMR pulse sequence with suppression of the water peak (NOESYPREAST) was used: RD – P(90°) – 4 µsec – P(90°) – tm – P(90°) – acquisition of the free induction decay (FID). The non-selective 90° hard pulse P(90°) was adjusted to 9.4 µsec. The relaxation delay (RD), the mixing time (tm), and the acquisition time were set to 4 sec, 100 msec, and 3.96 sec, respectively, resulting in a total recycle time of ~8.0 sec. Low-power continuous-wave irradiation on the water resonance at an field strength of ~25 Hz was applied during RD and tm for pre-saturation. After application of 4 dummy scans, 32 FIDs were collected into 32768 (32K) complex data points using a spectral width of 20.689 parts per million (ppm). FIDs were multiplied with an exponential function corresponding to a line broadening of 0.3 Hz before Fourier-transformation. Spectra were manually phase- and baseline-corrected and automatically referenced to the internal standard (TSP – 0.0 ppm) within TopSpin 1.3 (Bruker BioSpin).

*Metabolomics measurements: quality control and normalization of metabolite levels*

The Fourier-transformed and baseline-corrected NMR spectra were manually annotated by spectral pattern matching using Chenomx NMR Suite 6.1 (Chenomx Inc., Edmonton, Alberta, Canada) to deduce absolute urinary concentrations of 56 metabolites; subsequently, the NMR data was reduced to these metabolites. Similar to MS measurements, urinary dilution was accounted for by PQN normalization. Normalized metabolite levels were once more log_2_-transformed.

*Plasma measurements*

Plasma samples were stored frozen at -80°C until analysed. After thawing, 250 μL of plasma were mixed with 250 μL of phosphate buffer [prepared with D2O and contained sodium 3-trimethylsilyl-(2,2,3,3-D4)-1-propionate (TSP) as reference, (pH 7.4)].

Spectra were recorded on a Bruker AVANCE-II 600 NMR spectrometer operated by TOPSPIN 3.2 software (both Bruker Biospin, Rheinstetten, Germany), equipped with 5-mm z-gradient probe (Bruker Biospin, Rheinstetten, Germany) and automated tuning and matching (ATMA) unit (Bruker Biospin, Rheinstetten, Germany). Specimens were automatically delivered to the spectrometer via SampleJet (Bruker Biospin, Rheinstetten, Germany) into standard 5 mm NMR tubes. The acquisition temperature was set to 310°K. A standard one-dimensional ^1^H-NMR pulse sequence with suppression of the water peak (NOESYPREAST) was used. The sequence has the form –RD-gz,1-90°-t-90°-tm-gz,2-ACQ, where RD is the relaxation delay (4 sec) t is a short delay (~3 µsec), 90° represents the 90° RF hard pulse, tm is the mixing time (10 msec), gz,1 and gz,2 are the magnetic field z gradients both applied for 1 msec and ACQ is the acquisition period (2.7 sec) collecting 98304 data points at a sweep width of 30 ppm. The receiver gain is set at 90.5 for all experiments. For pre-processing, a line broadening of 0.3 Hz, a zero filling to produce 128k data points and a first-order phase correction of 0.0 was applied.

Spectral processing included zerofilling, linebroadening, Fourier transformation and referencing of the chemical shift and determination of the spectral intensity per 1 mmol protons for quantitative referencing. Chemical shifts of plasma spectra were referenced to the CH3-group signal of alanine adjusting it to 1.48 ppm. Spectra were segmented into N = 450 consecutive integrated spectral regions (buckets) of fixed width covering the region from 0.3 ppm to 1.4 ppm. Two subregions, i.e. 1.31-1.35 ppm and 1.16-1.21 ppm where excluded from binning, in order to avoid unwanted influences coming from lactate and ethanol CH3-group signals in later application of the method in typical population cohorts. Finally, the spectrum is submitted to data analysis for lipoprotein subclass analysis B.I.LISA^TM^ (Bruker BioSpin GmbH Germany).

For total, HDL- and LDL-cholesterol as well as total triglycerides comparison with laboratory measurements yielded excellent correlations (all above 0.91). However, six subjects revealed as outlier in PCA were excluded from the data set. Similar to the data above, all values were log_2_-transformed prior statistical analyses.

***Additional data processing***

*Data integration*

Most of the metabolites were unique to one of the applied techniques. However, 44 and 22 metabolites in plasma and urine, respectively, were overlapping with at least one other technique. With respect to plasma, following the grouping of metabolites in biochemical classes (i.e. lipids, amino acids and carbohydrates), correlations of those metabolites measured on both platforms were computed with all members of the same biochemical class. Subsequently, the metabolite with the higher median correlation across all class members was kept for further analysis. Regarding the lower sensitivity of NMR compared to MS, in cases of duplicated measures, those obtained on the MS were kept for further analyses. In total, 613 plasma and 587 urine metabolites were used in the subsequent statistical analyses.

To avoid spurious results in linear regression analysis, univariate outliers for each metabolite were excluded whenever concentrations exceeded more than three standard deviations from the mean value.

***Statistical analyses***

*Gaussian graphical model*

To facilitate integration of multifluid data we computed a Gaussian graphical model (GGM) because of its ability to reconstruct physiological dependencies purely in a data driven manner (5). This allowed us to evenly make assumption about the biochemical context of up to now unknown compounds. Briefly, a GGM relies on full-order partial correlations, which mean that a correlation between two metabolites only exists if it is independent from all remaining metabolites in the data set. Significant partial correlations after Bonferroni correction were visualized as network using Cytoscape 3.2.1. Since GGMs require a full data matrix missing values were imputed using the k-nearest-neighbor imputing as implemented in the R-package *impute*. K was set to ten and only metabolites with less than 20% missing values were considered (N=872). The final GGM comprised 785 nodes and 1065 edges.

***Replication Population SHIP-2***

SHIP (Study of Health in Pomerania) is based on representative samples of the population living in West Pomerania, a region in northeast Germany. The sampling was based on official data from population registries in the Federal State of Mecklenburg/West Pomerania. Study subjects received a maximum of three written invitations, followed by repeated telephone calls and in-person contacts at home. The baseline examinations in the SHIP cohort were performed between 1997 and 2001 with a total of 4308 men and women aged 20–79 years (response rate 68.8 %). The first follow-up (SHIP-1) was performed between 2002 and 2006 with 3300 subjects being re-examined (response rate 83.6 %). The second follow-up (SHIP-2) was performed between 2008 and 2012 with 2333 subjects being re-examined (response rate 62.9 %).

**Table S1** General Characteristics of the SHIP-2 population.

| **Characteristic** | **Men (n = 637)** | **Women (n = 782)** | **p*** |
| --- | --- | --- | --- |
| Age (years) | 55 (44; 65) | 53 (44; 64) | 0.24 |
| Smoking (%) |  |  | <0.01 |
| never smoker | 25.5 | 47.3 |  |
| former smoker | 24.6 | 21.9 |  |
| current smoker | 49.8 | 30.8 |  |
| Physically active (%) | 49.6 | 55.6 | 0.02 |
| Waist circumference (cm) | 95 (89; 103) | 83 (75; 93) | <0.01 |
| NT-proBNP (ng/L) | 41 (23; 94) | 79 (46; 135) | <0.01 |
| Triglycerides (mmol/L) | 1.75 (1.22; 2.51) | 1.28 (0.89; 1.88) | <0.01 |
| HDL-cholesterol (mmol/L) | 1.24 (1.03; 1.50) | 1.54 (1.32; 1.80) | <0.01 |
| LDL-cholesterol (mmol/L) | 3.38 (2.76; 4.00) | 3.29 (2.60; 3.95) | 0.03 |
| Total cholesterol (mmol/L) | 5.4 (4.7; 6.1) | 5.4 (4.7; 6.3) | 0.28 |
| eGFR (mL/min/1.73m²) | 89 (77; 99) | 89 (76; 101) | 0.39 |
| ALT (µkatal/L) | 0.48 (0.36; 0.65) | 0.31 (0.25; 0.40) | <0.01 |
| Ejection fraction (%) | 70 (63; 76) | 72 (67; 77) | <0.01 |

HDL=high density lipoprotein; LDL=low-density lipoprotein; NT-proBNP=N-terminal prohormone of brain natriuretic peptide; eGFR=estimated glomerular filtration rate; ALT=alanine aminotransferase; Continuous data are expressed as median (25th percentile; 75th percentile); nominal data are given as percentages. *χ2-test (nominal data) or Mann-Whitney-U test (interval data) were performed.

**FIGURES**


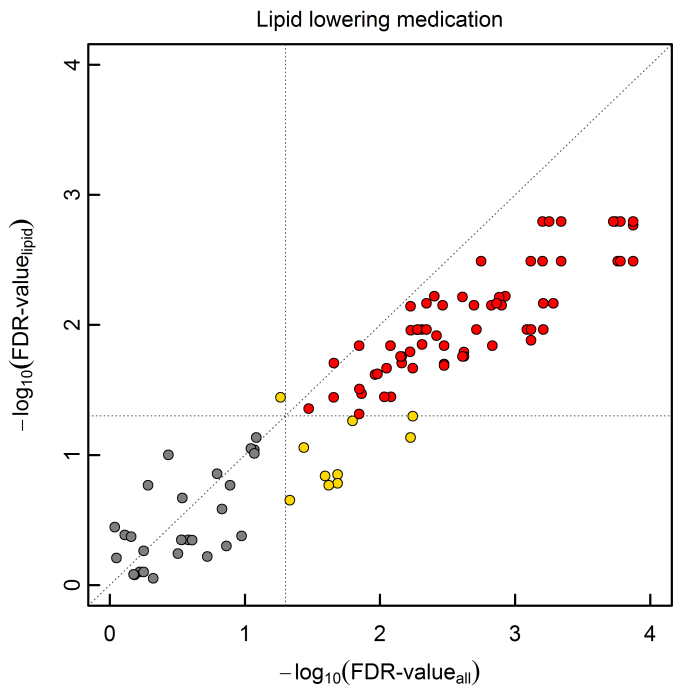


**Figure S1** Comparison of corrected p-values (false discovery rate - FDR) from linear regression analyses with serum NT-proBNP as exposure and lipoprotein subclasses as outcome either in the whole population (x-axis) or after exclusion of subjects taking lipid-lowering medication. Dots in red indicate lipoprotein measures significantly associated (FDR <0.05) in both samples, whereas yellow dots indicate significant associations in only one population.


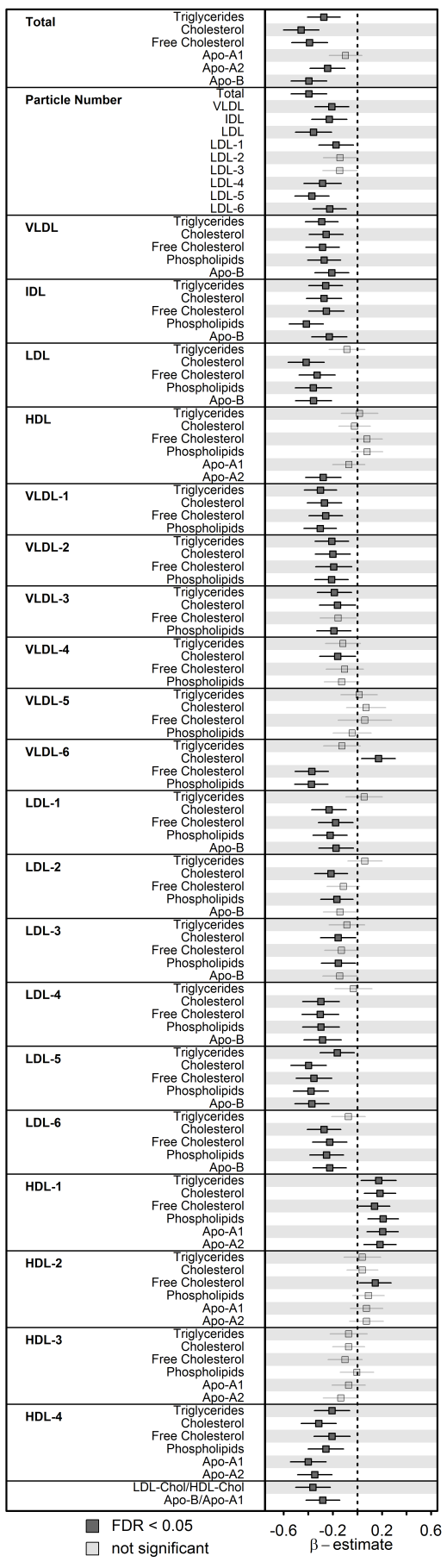
**Figure S2** Beta-estimates with 95%-confidence intervals from linear regression analysis for lipoprotein subclasses and derived variables with serum NT-proBNP concentrations in SHIP-2. Significant associations (controlling the false discovery rate (FDR) at 5%) are indicated by dark grey. VLDL = very low-density lipoprotein; IDL = intermediate-density lipoprotein; LDL = low-density lipoprotein; HDL = high-density lipoprotein; Apo = apolipoprotein

**References**

1. Evans AM, DeHaven CD, Barrett T, Mitchell M, Milgram E. Integrated, nontargeted ultrahigh performance liquid chromatography/electrospray ionization tandem mass spectrometry platform for the identification and relative quantification of the small-molecule complement of biological systems. Analytical chemistry. 2009;81(16):6656-67.

2. Lawton KA, Berger A, Mitchell M, Milgram KE, Evans AM, Guo L, et al. Analysis of the adult human plasma metabolome. Pharmacogenomics. 2008;9(4):383-97.

3. Dieterle F, Ross A, Schlotterbeck G, Senn H. Probabilistic quotient normalization as robust method to account for dilution of complex biological mixtures. Application in 1H NMR metabonomics. Analytical chemistry. 2006;78(13):4281-90.

4. Filzmoser P, Maronna R, Werner M. Outlier identification in high dimensions. Computational Statistics and Data Analysis. 2008;52(3):1694-711.

5. Krumsiek J, Suhre K, Illig T, Adamski J, Theis FJ. Gaussian graphical modeling reconstructs pathway reactions from high-throughput metabolomics data. BMC systems biology. 2011;5:21.
